# Forty years of hematopoietic stem cell transplantation in Croatia: First-World options and First-World science

**DOI:** 10.3325/cmj.2023.64.65

**Published:** 2023-04

**Authors:** Steven Z. Pavletic

**Affiliations:** National Cancer Institute, Center for Cancer Research, National Institutes of Health, Bethesda, MD, USA

In 1987, an article entitled Transplanted technology: Third World options and First World science was published in the *New England Journal of Medicine* (*NEJM*) (1). It referred to a medical miracle – a flourishing bone marrow transplant (BMT) program in Zagreb, which was approaching its fifth year of existence.

In this issue of the *Croatian Medical Journal*, Prof Labar describes the circumstances and significance of the achievements of the first hematopoietic stem cell transplant (HSCT) in Croatia 40 years ago (2). This procedure has not only saved the lives of numerous patients but has also transformed Croatian medicine through propelling the development of many accompanying clinical subspecialties required for the safe conduct of the transplant procedure. The Zagreb transplant team has been able to stay at the forefront of introducing the newest and latest technologies into their clinical practice. Impressively, this activity continued unshattered during the Croatian War for Independence, emphasizing the timeless value of human life and the indomitable human spirit.

The phenomenon of the Zagreb BMT program’s success was not a random occurrence or a result of the local political will. It would have been impossible without the strong scientific tradition of the Zagreb Immunology School centered around the Ruđer Bošković Institute and other research institutions (3-5). The pivotal role that Croatian immunologists played in laying the foundation for BMT was summarized in the seminal review article by Robert Truitt in 2002 (6). The Zagreb transplant team has also made a major impact on clinical science, as illustrated by the long list of articles in leading medical journals as is presented in the current article (2).

Today, more than 80 000 HSCTs are performed per year by more than 1600 teams in 86 countries. Almost two million HSCTs have been performed worldwide since its inception (7). All this reflects the immense impact of HSCT on public health and the progress in medicine.

The Zagreb team was at the helm of that effort in February 1983, when transplants in Croatia started. Where this timing fits in the general trajectory of the history of the science of HSCT is best illustrated in a 2007 *NEJM* editorial (8). As transplants have become safer and more accessible, the field has shifted focus from reducing early mortality to the prevention and treatment of those who are cured from leukemia but developed chronic graft-vs-host disease (GVHD), a late complication of HSCT (9,10).

However, the picture of this success would be incomplete without emphasizing the international recognition of this program, which started through the collaboration with the European School of Oncology in 1987 and the first international conference on the treatment of acute leukemia held in Dubrovnik. The second milestone was the training and education of the early-career Croatian hematologists in the best international medical centers including Fred Hutchinson Cancer Research Center in Seattle, University of Nebraska Medical Center in Omaha, Johns Hopkins University in Baltimore, and the National Cancer Institute in Bethesda. These efforts led to the establishment of the leading international Chronic GVHD multidisciplinary program in Zagreb in 2013 (10).

When it comes to HSCT, in 2023 the “Third World” and the “First World” have clearly become “one world.” The time has come for the next generation to turn the page and assume leadership for the next 40 years. We hope that leukemia and other lethal bone marrow diseases will be eliminated by then. Today, our gratitude and the gratitude of many patients and their families goes to our dedicated colleagues in Zagreb who pioneered the transplant program four decades ago.
